# Improved Variational Mode Decomposition and CNN for Intelligent Rotating Machinery Fault Diagnosis

**DOI:** 10.3390/e24070908

**Published:** 2022-06-30

**Authors:** Qiyang Xiao, Sen Li, Lin Zhou, Wentao Shi

**Affiliations:** 1 School of Artificial Intelligence, Henan University, Zhengzhou 450046, China; xqy@henu.edu.cn (Q.X.); lsien@henu.edu.cn (S.L.); 2 School of Marine Science and Technology, Northwestern Polytechnical University, Xi’an 710072, China

**Keywords:** intelligent fault diagnosis, improved variational mode decomposition, deep learning, continuous wavelet transform (CWT)

## Abstract

This paper proposes an intelligent diagnosis method for rotating machinery faults based on improved variational mode decomposition (IVMD) and CNN to process the rotating machinery non-stationary signal. Firstly, to solve the problem of time-domain feature extraction for fault diagnosis, this paper proposes an improved variational mode decomposition method with automatic optimization of the number of modes. This method overcomes the problems of the traditional VMD method, in that each parameter is set by experience and is greatly influenced by subjective experience. Secondly, the decomposed signal components are analyzed by correlation, and then high correlated components with the original signal are selected to reconstruct the original signal. The continuous wavelet transform (CWT) is employed to extract the two-dimensional time–frequency domain feature map of the fault signal. Finally, the deep learning method is used to construct a convolutional neural network. After feature extraction, the two-dimensional time-frequency image is applied to the neural network to identify fault features. Experiments verify that the proposed method can adapt to rotating machinery faults in complex environments and has a high recognition rate.

## 1. Introduction

Rotating machinery is widely used in large-scale manufacturing systems and important technical equipment, such as ships and oceans, wind turbines, aerospace engines, etc. [1,2,3]. In these systems and equipment, the key components in rotating machinery, such as rolling bearings and gears, often have early defects such as mild wear, spot eclipse, and so forth. Bearings are some of the most important components among them. The operating state of the rotating equipment is heavily dependent on the state of the bearings. If these defects are not diagnosed at the incipient stage, they will continue to deteriorate over time, eventually leading to system failure and causing considerable losses to people’s lives and properties [4,5]. Hence, to ensure the normal operation of rotating machinery, it is necessary to carry out fault diagnosis and health assessment of bearings.

The key to achieving fault diagnosis of bearings is to extract useful information related to fault characteristics from the analyzed signals. Vibration analysis-based methods have been studied for decades, and vibration has long been one of several main parameters in the fault diagnosis of rotating machinery [6]. The vibration signal collected by bearings contains very important dynamic characteristics, so it is very important and effective to analyze the collected vibration signal to identify its operating state [7,8,9]. However, the working environment of the bearings is complex, and its vibration signals are affected by various excitation sources, which often have continuous, nonlinear, and non-stationary characteristics [10,11]. This situation brings difficulties to signal analysis, feature extraction, and later work status identification. The fast Fourier transform (FFT) method is only suitable for stationary signal processing, while short-time Fourier transform, wavelet transform (WT), and other methods lack adaptability [12,13].

The widely used empirical mode decomposition (EMD) method produces unexplained negative frequency phenomena in the non-stationary signal analysis and mode decomposition. This method also has problems with end effects, envelope fitting, and mode mixing [14,15,16]. Zheng et al. [17] proposed a new signal decomposition method, namely extreme-point weighted mode decomposition (EWMD), for improving the accuracy of EMD. For non-stationary multi-component signals, a novel approach named RRP-RD has been developed in [18] for ridge detection based on the gathering of ridge portions in the time-frequency plane. Fourer et al. [19] proposed the ASTRES Toolbox for mode extraction of non-stationary multi-component signals. This method offers efficient tools for analyzing, synthesizing, and transforming any signal made of physically meaningful components. Variational mode decomposition is an adaptive decomposition algorithm by rigorous mathematical reasoning [20]. This method can convert the mode decomposition into a variational solution problem and determine the variational solution problem by iteratively searching for the optimal solution of the variational mode. Variational mode decomposition (VMD) has received increasing attention in the diagnosis of rolling element bearings. In the method of VMD, an optimal determination of decomposition parameters is the pivotal point. However, this method may have the problem of choosing the mode number *K*. The number of decomposed modes *K* needs to be set before the VMD starts processing the signal, and the accurate determination of *K* is significant to the modal influence after the signal is decomposed [21,22]. However, most *K* values are determined by human experience and are not rigorous enough due to a lack of criteria.

Aiming to solve the optimization problem of decomposition parameters in variational mode decomposition, Ni et al. [23] proposed a fault information-guided VMD (FIVMD) method for extracting the weak bearing repetitive transient. Li et al. [24] first used the envelope kurtosis maximum as an indicator to optimize and determine the mode number of VMD and introduced a novel method to realize the selection of the optimal IMF(s) of VMD, which contain fault information based on frequency band entropy (FBE). Liang et al. [25] presented a multi-objective multi-island genetic algorithm (MIGA) to optimize VMD parameters and apply it to feature extraction of a bearing fault. The above methods can well solve the parameter optimization problem of variational mode decomposition and avoid the defects of artificial selection. However, parameter selection is mostly based on the optimization algorithm, and the analysis and processing of the decomposed intrinsic mode functions (IMFs) are insufficient. Furthermore, these methods are not combined with popular machine learning algorithms (e.g., neural networks). Ye et al. [26] decomposed the original bearing vibration signal into several IMFs by the VMD method and reconstructed the signal by the feature energy ratio (FER). They calculated the multiscale permutation entropy of the reconstructed signal to construct multidimensional feature vectors. The vector is fed into the PSO-SVM classification model for automatic identification of different fault patterns of the rolling bearing.

However, the above methods have certain limitations for fault data in the context of big data [27]. Thus, deep learning methods have emerged for fault diagnosis of bearings. A one-dimensional convolutional neural network has been established to diagnose bearing faults based on a data-driven diagnostic model. However, this method is mainly aimed at one-dimensional time-domain vibration signals and cannot fully extract the time–frequency-domain features of faults [28,29]. Hence, a two-dimensional convolutional neural network was established to avoid the one-dimensional convolutional neural network defects. In this method, the fault signal is spliced and processed and then converted into a two-dimensional image and input to the convolutional neural network for fault diagnosis [30,31]. However, this method does not process the time-domain feature of the fault signal, and there will be some redundant components in the original signal, which will affect the diagnosis accuracy.

This paper proposes an intelligent bearing fault diagnosis method based on the improved VMD and CNN. The technique uses the minimum average Pearson coefficient principle to analyze the fault signal in the time domain. The purpose is to determine the optimal mode number *K* for signal decomposition to extract fault features to maximize the difference between adjacent frequency bands and achieve the best decomposition effect. Then, the correlation analysis is performed on each component of the decomposed signal, and the component with a large correlation coefficient is selected for reconstruction. This method can remove the interference of redundancy in the signal and retain the main features. The CWT is used to transform the time-domain signal into a two-dimensional time–frequency image. After that, the preprocessed two-dimensional time–frequency image is applied to the convolutional neural network. Finally, the convolutional neural network adaptively extracts the time–frequency image features to achieve end-to-end fault diagnosis. The intelligent fault diagnosis model can be applied to the background of big data and has a good application effect.

## 2. Improved Variational Mode Decomposition

### 2.1. VMD Theory

The VMD algorithm can simultaneously decompose the vibration signal into multiple single-component AM (Amplitude Modulation) and FM (Frequency Modulation) signals, and the decomposition process is the solution process of the variational problem [20]. The objective of the VMD algorithm is to decompose the original signal into a specified number of band-limited intrinsic mode function (BLIMF) components by constructing and solving the constrained variational problem. The Gaussian smoothing index estimates the bandwidth of each component, and the optimal variational mode is established according to the constraints as follows:

The Variational Mode Decomposition (VMD) algorithm can decompose the vibration signal into multiple mode components.

(1) $$\left\{\begin{array}{l} \underset{\left\{u_{k}\right\},\left\{\omega_{k}\right\}}{\min}\left\{\sum_{k=1}^{K}\|\partial_{t}\left[\left(\delta \left(t\right)+\frac{j}{\pi t}\right)*u_{k}\left(t\right)\right]e^{-j\omega_{k}}{\|}_{2}^{2}\right\}\\ s.t.\sum_{k=1}^{K}u_{k}\left(t\right)=S\left(t\right) \end{array}\right.$$

In Equation (1), $u_{k}\left(t\right)$ is the narrow band mode function.$\left\{u_{k}\right\}:=\left\{u_{1},\ldots ,u_{K}\right\}$ and $\left\{\omega_{k}\right\}:=\left\{\omega_{1},\ldots ,\omega_{K}\right\}$ are shorthand notations for the set of all modes and their center frequencies, respectively. *K* is the number of BLIMFs that should be set before decomposition. Equally, $\sum_{k=1}^{K}u_{k}(t)$ is understood as the summation over all modes. $\delta \left(t\right)$ is the unit pulse function, and $e^{-j\omega_{k}}$ is an exponential term to adjust the center frequency corresponding to each modal component. $\partial_{t}$ is the partial derivative of $u_{k}\left(t\right)$ with respect to t. Furthermore, ∗ is the convolution operation symbol. S(t) is the sum of the components of the original input signal.

The optimal selection of $u_{k}\left(t\right)$ is obtained by solving the constrained variational problem given in Equation (1). The augmented Lagrange function is introduced to deal with the constrained variational problem.

(2) $$\begin{array}{c} L\left(\left\{u_{k}\left(t\right)\right\},\left\{\omega_{k}\right\},\lambda \left(t\right)\right)=\alpha \sum_{k=1}^{K}\|\partial_{t}\left[\left(\delta \left(t\right)+\frac{j}{\pi t}\right)*u_{k}\left(t\right)\right]e^{-j\omega_{k}t}{\|}_{2}^{2}\\ +\|S\left(t\right)-\sum_{k}^{K}u_{k}\left(t\right){\|}_{2}^{2}+\langle \lambda \left(t\right),S\left(t\right)-\sum_{k=1}^{K}u_{k}\left(t\right)\rangle \end{array}$$

where α is the quadratic penalty factor, and $\lambda \left(t\right)$ is the Lagrange multiplier.

The saddle point of the augmented Lagrange expression, the optimal solution of Equation (1), can be found by using the alternating direction multiplier algorithm (ADMM) to update alternately. The generating BLIMF components and their corresponding center frequencies $\omega_{k}$ are updated in the ADMM algorithm, which are represented as follows:

(3) $${\overset{\wedge }{u}}_{k}^{n+1}\left(\omega \right)=\frac{\overset{\wedge }{S}\left(\omega \right)-\sum_{i\neq k}^{K}{\overset{\wedge }{u}}_{i}\left(\omega \right)+\frac{\overset{\wedge }{\lambda }\left(\omega \right)}{2}}{1+2\alpha {\left(\omega -\omega_{k}\right)}^{2}}$$

(4) $$\omega_{k}^{n+1}=\frac{{\int_{0}^{\infty }\omega \left|{\overset{\wedge }{u}}_{k}\left(\omega \right)\right|}^{2}d\omega }{{\int_{0}^{\infty }\left|{\overset{\wedge }{u}}_{k}\left(\omega \right)\right|}^{2}d\omega }$$

Lagrange multiplier $\overset{\wedge }{\lambda }$ is updated by Equation (5), and *n* is the number of iterations.τ is the update parameter.

(5) $${\overset{\wedge }{\lambda }}^{n+1}\left(\omega \right)={\overset{\wedge }{\lambda }}^{n}\left(\omega \right)+\tau \left(\overset{\wedge }{S}\left(\omega \right)-{\sum_{k}^{K}\overset{\wedge }{u}}_{k}^{n+1}\left(\omega \right)\right)$$

The update process is stopped repeatedly until the condition is satisfied, as shown in Equation (6).

(6) $${\sum_{k}^{K}\left\|{\overset{\wedge }{u}}_{k}^{n+1}-{\overset{\wedge }{u}}_{k}^{n}\right\|}_{2}^{2}/{\left\|{\overset{\wedge }{u}}_{k}^{n}\right\|}_{2}^{2}<\varepsilon$$

ε is the accuracy. Thus, the signal can be adaptively decomposed into different mode signals.

### 2.2. Improved Variational Mode Decomposition

The collected one-dimensional bearing vibration signal is continuous-time and non-stationary in this paper. Before processing the signal by the VMD, it is necessary to set the *K* of the decomposed components. The accurate determination of *K* is very important for the mode effect after decomposing the signal. However, in the traditional VMD method, the determination of the mode number *K* is mostly based on experience, which has certain limitations. To solve the problem, this paper proposes the minimum average Pearson correlation coefficient principle to determine the optimal *K* for variational mode decomposition. This method expresses the degree of correlation between two adjacent components after decomposition by calculating the Pearson correlation coefficient. The Pearson correlation coefficient is defined as the quotient of the covariance and standard deviation between two variables. The smaller the value, the greater the difference between the two decomposition components. It also indicates that the effect of signal decomposition has been improved. The equation of the Pearson correlation coefficient is:

(7) $$\rho \left(X,Y\right)=\frac{\mathrm{cov}\left(X,Y\right)}{\sigma_{X}\sigma_{Y}}=\frac{E\left[\left(X-\mu_{X}\right)\left(Y-\mu_{Y}\right)\right]}{\sigma_{X}\sigma_{Y}}$$

where $\mathrm{cov}\left(X,Y\right)$ is sample covariance, $E\left[\left(X-\mu_{X}\right)\left(Y-\mu_{Y}\right)\right]$ is the mathematical expectation, $\sigma_{X}$ is the sample standard deviation of *X*, and $\sigma_{Y}$ is the sample standard deviation of *Y*.

Variational modal processing is performed on the bearing fault signal to obtain the decomposed components. The Pearson coefficient can be used to determine the correlation between the decomposed components and the original signal. According to the decomposition value *K* of the mode number and the equation of the Pearson coefficient, the equation of the average Pearson correlation coefficient is proposed in this paper as follows:

(8) $${\bar{\rho }}_{I_{i},I_{i+1}}=\frac{1}{K-1}\sum_{i=1}^{K-1}\rho \left(I_{i},I_{i+1}\right)$$

In the equation, $I_{i}$ and $I_{i+1}$ represent each BLIMF component after signal decomposition. *K* is the number of modes of the variational mode decomposition. For different *K* values, the algorithm steps are as follows:

1. Determine the iteration interval of the *K*.
2. In the iterative interval, an improved VMD decomposition is carried out on the vibration signals under different *K* values.
3. Calculate the Pearson correlation coefficient of each adjacent frequency band component decomposed separately by each *K* value.
4. After setting the *K*, calculate the sum of *K*−1 correlation coefficient values and obtain the average calculation for the fault signal component. Calculate the ${\bar{\rho }}_{I_{i},I_{i+1}}$.
5. The average Pearson coefficient values correspond to different *K* values, and the *K* corresponding to the smallest ${\bar{\rho }}_{I_{i},I_{i+1}}$ is the optimal number of decompositions.

## 3. Time–Frequency Feature Extraction

The collected one-dimensional bearing vibration signal can be converted into a two-dimensional time–frequency image by CWT and then input into the convolutional neural network to realize fault classification. The two-dimensional time–frequency image contains time-domain information and reflects the frequency-domain information of mechanical fault signals. Therefore, this paper reconstructs the extracted main mode components and uses continuous wavelets to extract the two-dimensional time–frequency image of mechanical faults.

### 3.1. Data Augmentation

**Figure 1:**
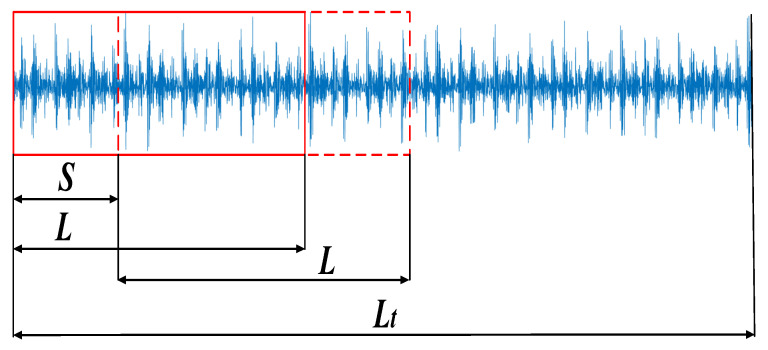
Oversampling and data augmentation.

In practical fault identification applications, there is usually a problem of insufficient fault data. These result in poor generalization performance of the diagnostic model. This paper adopts the method of overlapping sampling for the time series signal to increase the number of training samples. As shown in Figure 1, the frequency is 12 kHz, and the sampling time is 10 s. Therefore, the sampling signal contains a total of 120,000 data points. By overlapping sampling of the fault signal, a single sample length of 1000 data points is selected, the moving step is set to 100, and the calculation is as follows:

(9) $$N\leq \frac{L_{t}-L}{m}+1$$

It can be calculated that the total number of training samples is 1192. In this paper, 800 samples were randomly selected as the samples of each fault type. In the equation, *N* is the number of samples, The red square represents a single sampling signal. *L_t_* is the total length of the sampled signal, *L* is the length of a single sample signal, and *m* is the step size of each movement. The schematic diagram of overlapping sampling of signals is shown in Figure 1.

### 3.2. Continuous Wavelet Transform (CWT)

The one-dimensional vibration signal can be converted into a two-dimensional energy spectrogram by the method of CWT. For energy signal $y\left(t\right)$, the mathematical expression after CWT is as follows:

(10) $$WT_{f}\left(a,b\right)=\frac{1}{\sqrt{a}}\int {}_{-\infty }^{+\infty }y\left(t\right)\psi_{a,b}\left(\frac{t-b}{a}\right)dt$$

where $WT_{f}\left(a,b\right)$ is the wavelet coefficient, and $\psi_{a,b}\left(t\right)=\frac{1}{\sqrt{a}}\psi \left(\frac{t-b}{a}\right)$ is the wavelet base function that depends on *a* and *b*. $\psi \left(\frac{t-b}{a}\right)$ is the mother wavelet function, *a* > 0 is the scale factor, and *b* is the translation parameter. The waveform of the Morlet wavelet is similar to the vibration signal fault shock waveform of the bearing. Furthermore, the cmor wavelet is the complex form of Morlet wavelet and has good adaptive performance. In this paper, the cmor wavelet was chosen as the wavelet basis function. Its bandwidth is 3 and the wavelet center frequency is 3 Hz. The scale sequence length is 256.

## 4. Mechanical Rotation Fault Diagnosis Based on Convolutional Neural Network

**Figure 2:**
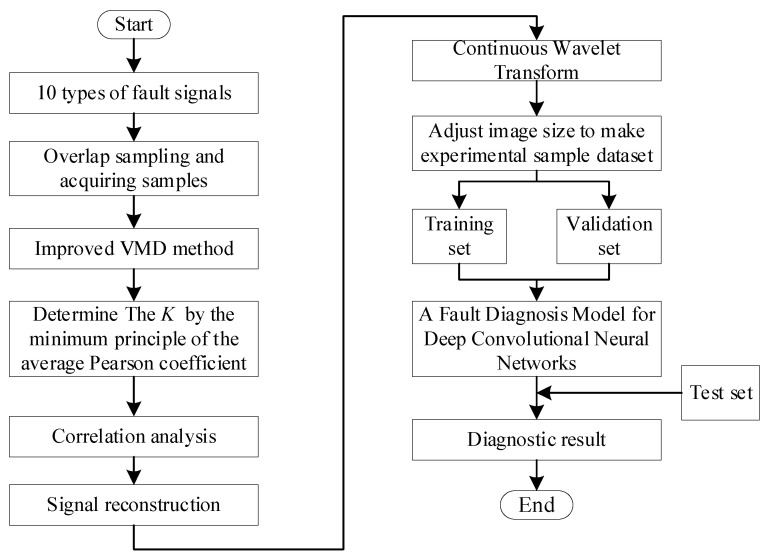
Flow chart of intelligent bearing fault diagnosis model based on improved VMD.

At present, bearing fault diagnosis faces several problems, such as the background of big data and the limitations of traditional fault diagnosis. An IVMD method based on the minimum average Pearson coefficient principle is proposed to determine the optimal *K* for signal decomposition. The deep learning method is used to establish a convolutional neural network to extract fault features and use this network to identify and classify various fault types. The neural network is employed to perform multi-layer nonlinear learning on the preprocessed vibration signal, which can automatically extract fault features and diagnose the health status of the system. The flow chart of the established intelligent diagnosis model is presented in Figure 2.

### 4.1. Convolutional Neural Network

The CNN is a special NN with a feed-forward structure. The CNN has three important characteristics that make its strength in 2-D image analysis, including local receptive fields, weight sharing, and sub-sampling in the spatial domain. A typical 2-D CNN consists of three major layers: convolutional layer, pooling layer, and fully-connected layer. After several alternating convolutional and pooling layers, the fully connected layers are followed to compute the class scores.

The convolutional layer applies a set number of kernels to obtain the feature maps of input images. The convolution layer can be described using the following equation:

(11) $$x_{j}^{l}=f\left(\sum_{i\in M_{j}}x_{i}^{l-1}*k_{ij}^{l}+b_{j}^{l}\right)$$

where $x_{j}^{l}$ denotes the output of the *j*th neuron in layer *l*. ∗ is the convolution operation. *M_j_* is a selection of input maps. *l* is the *l*th layer in the network.$k_{ij}^{l}$ and $b_{j}^{l}$ denote the weights and bias of the convolution kernel *i*th convolution kernel in layer *l,* respectively.f(·) is a nonlinear activation function. The rectified linear unit (ReLU) is used as the activation function between the convolution layers in this paper. It can be described using the following equation:

(12) $$f\left(x\right)=max\left(0,\mathrm{lg}\left(1+e^{x}\right)\right)$$

where *x* represents the output of the convolution layer.

The pooling layer is often connected after the convolutional layer to reduce the dimension of the feature map and decrease the number of parameters in the network. The max-pooling function can be described as the following equation:

(13) $$x_{j}^{i}=f\left(\beta_{j}^{l}down\left(x_{i}^{l-1}\right)+b_{j}^{l}\right)$$

where $x_{j}^{l}$ denotes the output of the *j*th feature map in layer *l*. down(·) is the max-pooling function in this paper. Each output map is given its own multiplicative bias β and additive bias *b*.

After the input image is transmitted alternately through multiple convolution and pooling layers, the extracted features are classified using the full connection layer network. The Softmax function is used as an activation function for the output layer in this paper. The loss function of the CNN is the cross-entropy between the estimated Softmax output probability distribution and the target class probability distribution. The cross-entropy function was used in our paper. Furthermore, the Adam Stochastic optimization algorithm was applied to train the CNN to minimize the loss function.

### 4.2. Deep Learning-Based Rotating Machinery Fault Diagnosis Model

**Figure 3:**
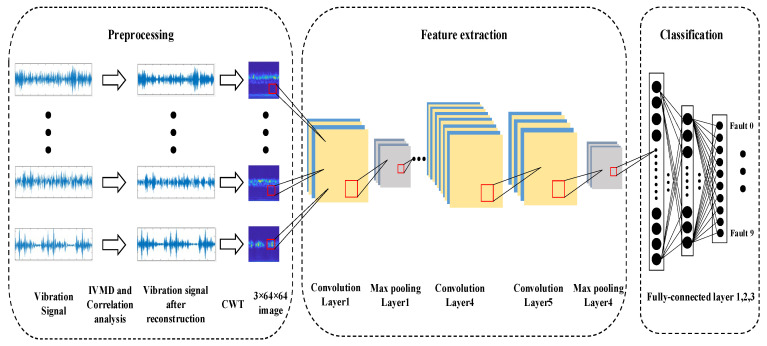
The model of IVMD-CNN for bearing fault diagnosis.

The bearing fault diagnosis model based on the IVMD and CNN methods is shown in Figure 3. The method developed in this paper consists of three main parts.

(1) Preprocessing.

Step 1: Select 10 types of fault signals in the dataset. 

Step 2: The optimal decomposition number *K* of these fault vibration signals is determined by the average Pearson principle proposed in this paper.

Step 3: These signals are decomposed by the IVMD method, and the decomposed components are subjected to correlation analysis and reconstruction.

Step 4: Convert the one-dimensional signals into two-dimensional time–frequency images by CWT.

Step 5: Resize these images to 3 × 64 × 64.

(2) Feature extraction.

Step 6: Establish a CNN to train the preprocessed fault image dataset, and the network is used to extract the image features of these fault signals.

(3) Classification.

Step 7: Fully connected layers are used to classify different types of faults. The model is established to complete the task of fault diagnosis.

## 5. Experiment

We used the signal processing toolbox in MATLAB (R2019a), and the CNN model was built on Python 3.7 with PyCharm. The experiment was applied on a computer with an Intel Core I5 7th generation CPU and 16 GB memory.

### 5.1. Preprocessing

#### 5.1.1. Test-Rig Description

In this paper, the real bearing data were used to evaluate the performance of the proposed method. The data were obtained from the Case Western Reserve University Bearing Fault Database [32,33], one of the benchmark datasets for bearing diagnosis. This dataset uses EDM technology to process pit defects on the inner ring, outer ring, and rolling elements of rolling bearings to simulate faults and collect vibration signals in different states. The vibrations are collected for about 10 s with sampling frequencies at 12 kHz and 48 kHz. The dataset has four different types of health states including the normal type, outer ring fault (OR), inner ring fault (IR), and rolling element fault (Ball). Except for the normal type, each fault type contains three different damage sizes. They are 0.18 mm, 0.36 mm, and 0.54 mm. Therefore, the dataset contains 10 different fault types.

In this experiment, 12 kHz was chosen as the sampling frequency, and the vibration signal of the driving end under working condition 1 was used for the experiment. The training of the fault model adopted a supervised training method. By labeling the ten status signals, the fault data parameters were obtained, as shown in Table 1.

#### 5.1.2. Iteratively Determine the Optimal *K*

The IVMD decomposed the fault signal to obtain a series of components. The iteration range of *K* was set to 5–10, and the average Pearson coefficient value corresponding to *K* in the iteration range was calculated.

**Figure 4:**
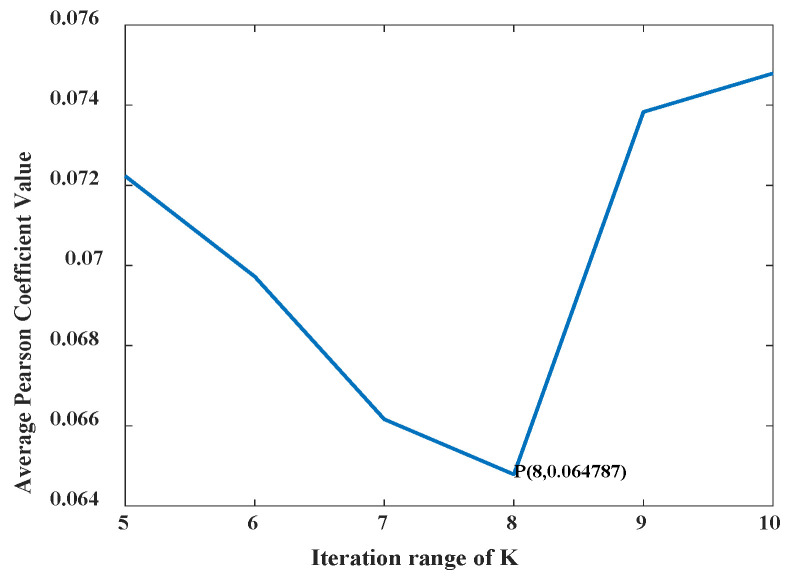
Average Pearson coefficient variation diagram of fault 6.

The optimal number of mode decomposition *K* values under different fault states was determined according to the minimum average Pearson coefficient principle. This condition can maximize the difference of signals in adjacent frequency bands to obtain the best decomposition effect of bearing fault signals after IVMD decomposition of signals in different fault types. In this paper, different fault types under working condition 1 were selected to verify the performance of the diagnostic model. By the IVMD decomposition method, the average Pearson coefficient corresponding to different *K* values was obtained in each fault type. The corresponding minimum *K* in each state was selected as the optimal number of decomposition modes. As an example, a line graph of the average Pearson coefficient value in the iteration interval *K* = 5–10 is shown in Figure 4 for fault 6.

According to the average Pearson coefficient values in Figure 4, the minimum average Pearson coefficient was 0.0648, the corresponding *K* was 8, and the fault signal decomposition effect could achieve the best effect. The average Pearson coefficient value was calculated for the other nine fault signals in the iterative interval, as shown in Table 2.

#### 5.1.3. Robustness Evaluation

**Figure 5:**
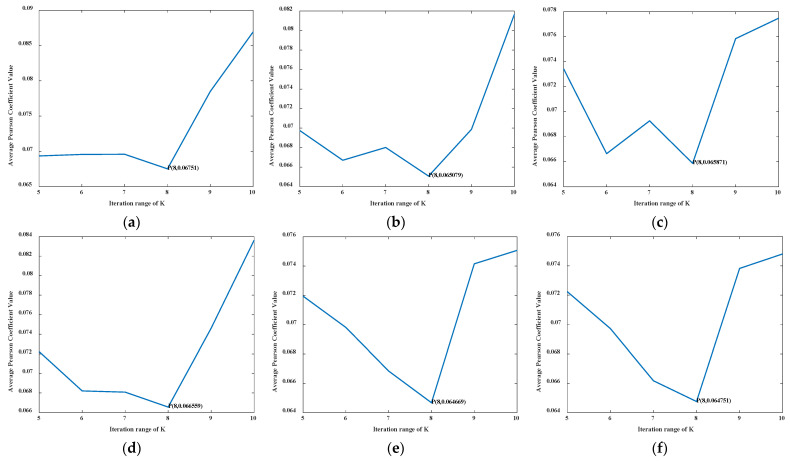
Results of different noise conditions: (**a**) *SNR* = 5 dB; (**b**) *SNR* = 10 dB; (**c**) *SNR* = 15 dB; (**d**) *SNR* = 20 dB; (**e**) *SNR* = 30 dB; (**f**) *SNR* = 50 dB.

The proposed IVMD in this paper was based on the method of minimum average Pearson coefficient. We provided a method to automatically select the mode number *K* of the VMD. To evaluate the robustness of the IVMD, Gaussian white noise (GWN) was added to signal to mimic the conditions with different signal-to-noise ratios (*SNR*s). The *SNR* is defined according to the following equation:

(14) $$SNR=10\log_{10}\left(\frac{P_{signal}}{P_{noise}}\right)$$

where $P_{signal}$ is the power of the signal, and $P_{noise}$ is the power of the noise. As in the selection in Section 5.1.2, the segment signal of fault 6 was selected for evaluation, and the results are shown in Figure 5.

Figure 5 shows the results of the proposed method with different *SNR* values in decibels (dB). The *K* determined by this method was invariant at different *SNR* values. The number of components determined was *K* = 8. The results in this experiment showed that the proposed method has certain robustness and can work with noisy vibration signals.

#### 5.1.4. Improved VMD Decomposition

**Figure 6:**
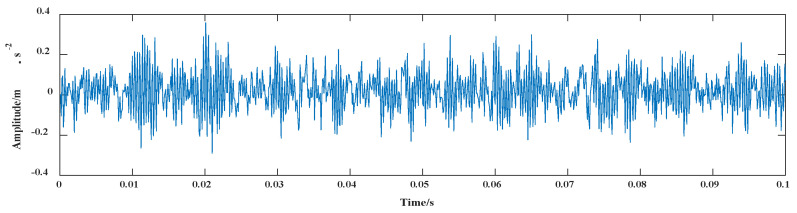
Vibration waveform of fault 6 signal.

In this paper, the segment signal was randomly selected in the original signal of fault 6, and the time-domain display is depicted in Figure 6.

**Figure 7:**
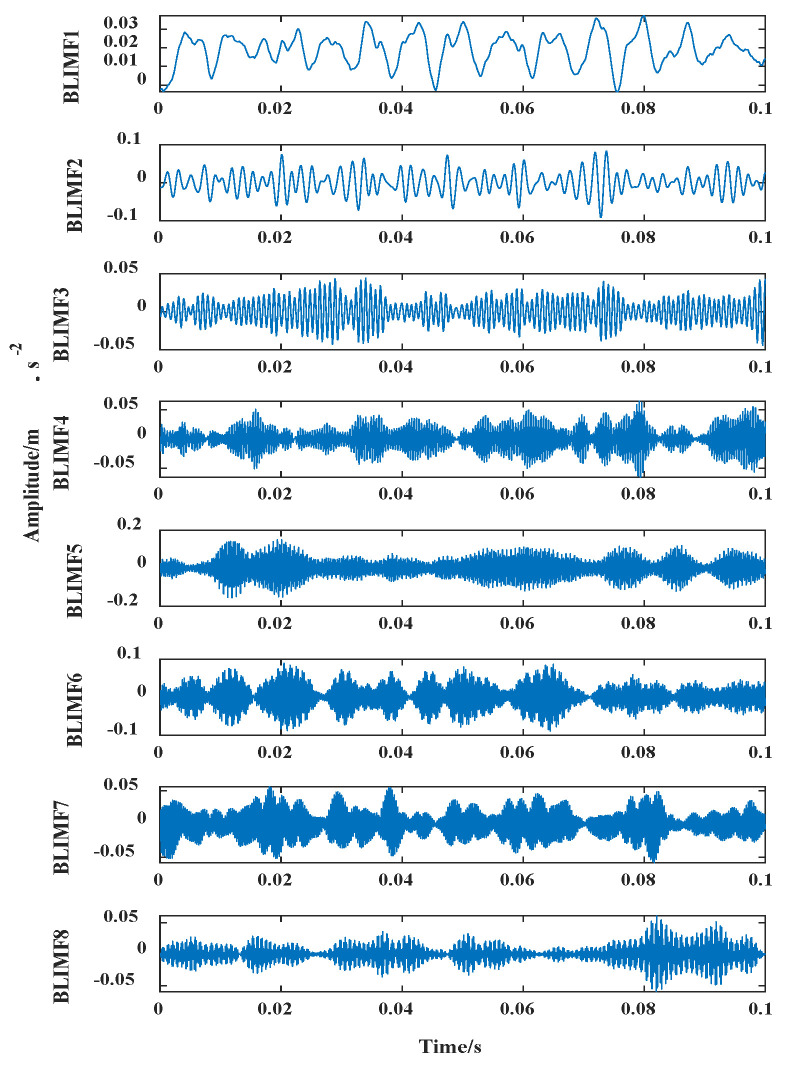
Improved VMD decomposition diagram.

For this period of the time-domain vibration signal, the average Pearson coefficient of the iterative interval was calculated using the IVMD method. Finally, the number of decomposed modes was 8. The vibration waveforms of BLIMF1–8 components after the decomposition of fault 6 are shown in Figure 7.

The IVMD method used in this paper could maximize the difference of BLIMF1–8 in adjacent frequency bands after decomposition to achieve the best decomposition effect for the fault 6 signal.

### 5.2. Feature Extraction

#### 5.2.1. Correlation Analysis

Correlation represents the degree of correlation between two variables that changes in the range of −1 to 1. A positive correlation value indicates that the two variables are positively correlated. Conversely, a negative correlation coefficient indicates that the two vectors are negatively correlated. The analysis showed that the vibration signal had continuity and accuracy, and the components after decomposition remained independent. This paper used the Pearson correlation coefficient method to analyze the fault signal components. The specific method was to find the correlation coefficient between each component after determining the optimal decomposition quantity of *K*. For two independent variables *X* and *Y*, the Pearson correlation coefficient could be calculated by Equation (7).

#### 5.2.2. Signal Reconstruction

This section analyzes the correlation between the fault signal components after determining the *K* and the original signal. The correlation coefficient between each decomposed component and the original signal was calculated, and the coefficient was used as the criterion to judge whether the mode component was the main component. The difference between this method and the method presented in the previous section is that the principle adopted in this section was to select a series of decomposed BLIMF components with a more significant correlation coefficient between the signal components and the original signal. The BLIMF components with a correlation coefficient greater than 0.3 were selected for reconstruction analysis. The correlation analysis of the fault 6 signal, as an example, is shown in Table 3. Using the equation of the Pearson coefficient, the correlation calculation between the eight decomposition components of fault 6 and the original signal could be obtained, as shown in Table 3.

**Figure 8:**
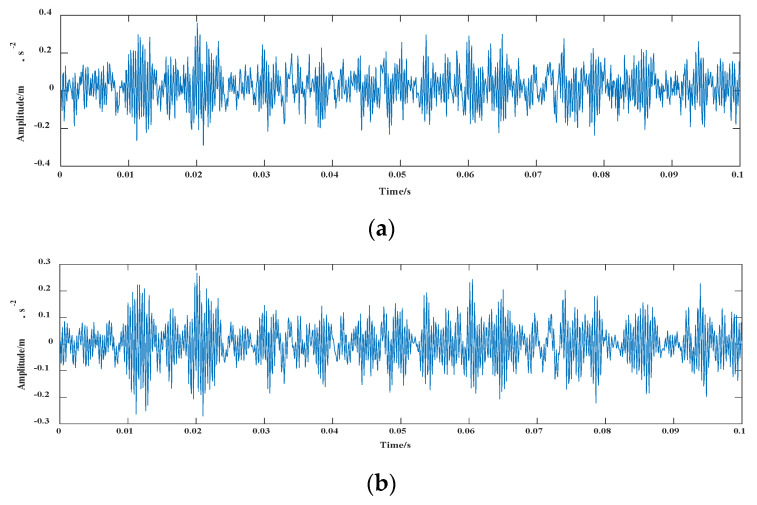
Vibration waveforms of fault 6 signal: (**a**) vibration waveform before reconstruction; (**b**) vibration waveform after reconstruction.

**Figure 9:**
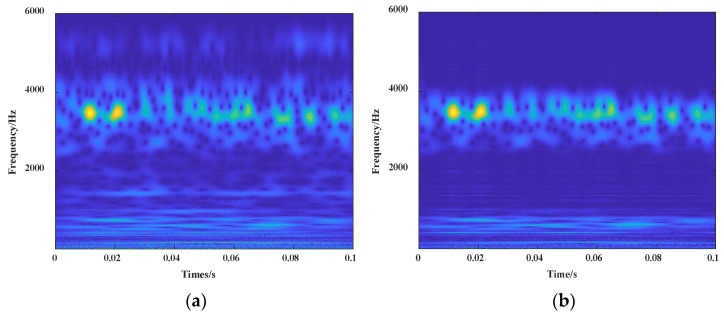
CWT images of segmented signal of fault 6: (**a**) CWT image before reconstruction; (**b**) CWT image after reconstruction.

In this section, components with correlation coefficients greater than 0.3 were selected to reconstruct BLIMF2, 4, 5, and 6. The signal comparison before and after the processing of signals are shown in Figure 8 and Figure 9.

The same method was used to perform correlation analysis on the signals of the other 9 faults and to select the reconstructed components to obtain the reconstruction information shown in Table 4.

The main mode components in the original signal were calculated by the correlation coefficient analysis method. Each component was extracted for reconstruction, the redundant information in the fault signal was removed, and the main features were retained. The reconstructed signals of different fault types were subjected to CWT. The fault information was analyzed in the time–frequency domain to realize the time–frequency-domain feature extraction of the fault signal.

#### 5.2.3. Decomposition Quality Evaluation

IVMD was compared with the EMD and original VMD (the original VMD method proposed in Ref. [20] with the mode number set to 5, since *K* = 5 is the default value when using the VMD method in MATLAB [34] in simulation and experimental datasets). In order to compare the decomposition performance of the three methods, orthogonal index (*OI*) and root mean squared error (RMSE) were considered as evaluating indicators. The *OI* is defined using the following equation in Ref. [14]:

(15) $$OI=\sum_{t=0}^{T}\left(\sum_{j=1}^{n+1}\sum_{k=1}^{n+1}C_{j}\left(t\right)C_{k}\left(t\right)/X^{2}(t)\right)$$

where *T* is the length of the BLIMF; *n* is the number of BLIMFs; *C_j_*(*t*) and *C_k_*(*t*) are the *j*th and *k*th BLIMF, respectively; and *X*(*t*) is the original signal. A smaller *OI* indicates better decomposition results. The RMSE definition is as follows in this paper:

(16) $$RMSE=\sqrt{E{\left(o\left(t\right)-r\left(t\right)\right)}^{2}}$$

where *o*(*t*) and *r*(*t*) are the original signal and reconstructed signal, respectively. The *RMSE* value should be zero. In theory, an *RMSE* value closer to zero indicates that the obtained reconstructed signals are closer to the original signal. As in the selection in Section 5.1.2, the segment signal of fault 6 was selected for IVMD and original VMD and EMD analysis, and the evaluation results of the reconstructed components are shown in Table 5.

The comparison results of three methods are shown in Table 5. It can be seen that the *RMSE* and *OI* of IVMD were smaller than those of the original VMD and EMD, which means that the reconstructed signal was more approximate to the original signal, and BLIMF components had smaller mode mixing problems.

### 5.3. Intelligent Fault Diagnosis

#### 5.3.1. Making the Dataset

For bearing faults, a suitable diagnostic model was established using CNN, and a dataset was made to train the hyperparameters in the neural network. As can be seen in the previous section, for each fault type signal, the number of training samples could be obtained using the data augmentation method. A total of 8000 samples was randomly selected as the sample set for this type of fault diagnosis. Then, ten kinds of fault datasets, including 8000 sample sets, were randomly divided into 7000 samples for the training set, 250 samples for the testing set, and 750 samples for the validation set. Ten different types of fault diagnosis images were obtained from the preprocessed time-domain data through CWT. The fault images were enhanced with random angle rotation, flip, scale transformation, and translation before inputting into the neural network. The processed time-domain signal was converted into a 64 × 64 × 3 RGB time–frequency image dataset. For different types of fault signals, each sample of the same fault type was labeled after conversion, the image training set was scrambled, and the images were input into the neural network for identification and classification by supervised training.

#### 5.3.2. CNN Parameters

The network had five convolutional layers, four max pooling layers, and three fully-connected layers. The parameters of each layer are shown in Table 6.

In the convolutional neural network, the step sizes of the convolution kernel traversal parameter and the padding were set to 1. Moreover, the convolution kernel size was 3. The kernel size and stride of the max-pooling layer were set to 2, and padding was set to 1. The number of output classifications for the last layer was 10. The diagnostic model adopted a supervised training method. During preprocessing of the dataset, the signals of different fault types were labeled. The learning rate was 0.0001. Dropout was used in the training process, which was set to 0.5 in the fully connected layer, which means that half of the neurons were randomly deactivated. The dropout layer could avoid falling into the local optimal solution problem during the iterative process of the diagnostic model and could also improve the accuracy and generalization performance of the diagnostic model.

#### 5.3.3. Training and Testing of the Diagnostic Model

**Figure 10:**
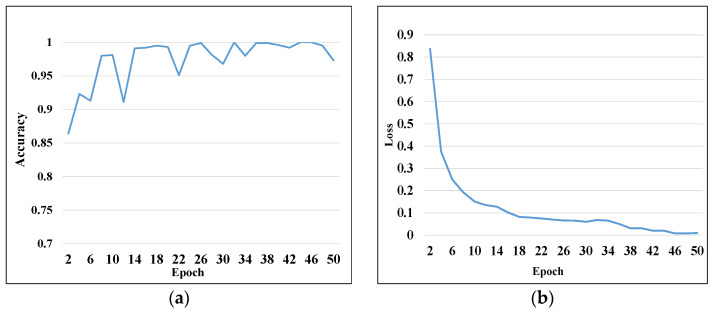
Training results of 50 epochs: (**a**) training accuracy results; (**b**) training loss results.

The neural network was fed by randomly scrambled samples consisting of 7000 samples in the training set and 750 samples in the validation set. The parameters of the convolutional neural network were set, and the network was trained with the mini-batch method. The size of the mini-batch was 32, and the training epoch was required to process 219 batches. When all training batches were completed, one iteration cycle of training ended, and the number of neural network training epochs was set to 50. The results of the recognition accuracy curve and loss function curve after model training are shown in Figure 10.

**Figure 11:**
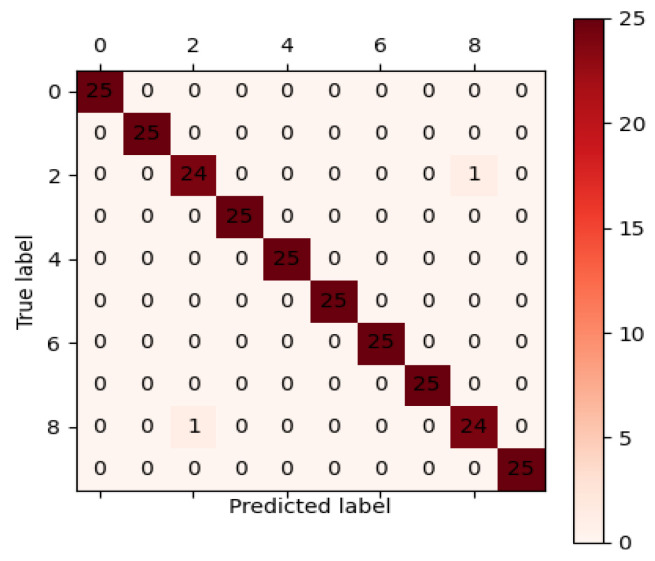
Confusion matrix of test set classification results.

As can be seen in the training results, the recognition accuracy of the proposed diagnostic model tended to be stable after 15 epochs of training. However, the loss function curve did not converge to the minimum, and the backpropagation of the neural network continued. Thus, the loss of the model continued to converge to the minimum. The next stable point for recognition accuracy of the model was after 38 epochs, which had the highest recognition accuracy of the model, equal to 97.3%. Moreover, the average training accuracy of 30 epochs was 97.10%. After 50 epochs of training results, the average recognition accuracy of the diagnostic model was 97.12%, and the loss function convergence was stable at around 0.009. Hence, to avoid overfitting of the diagnostic model, the results after 50 epochs of training were used as the diagnostic model for the test set. The parameters of the diagnostic model after 50 rounds of training were saved, and 250 samples of the test set were applied to the neural network to identify and classify different faults. The confusion matrix that expresses the prediction results of the test set sample recognition is illustrated in Figure 11.

The vertical axis of this matrix represents the true labels of fault types, and the horizontal axis shows the predicted fault type labels identified after applying the model. Analysis shows that the diagonal line of the matrix represents the number of correctly classified samples, and other blocks represent the number of samples that are wrongly classified into other categories. As can be obtained from Figure 10, the recognition accuracies of fault 2 and fault 8 were 96%, and the recognition accuracy of other fault types was 100%. Additionally, the average recall for this model was 99.2%. The analysis shows that the diagnostic model had higher fault recognition and higher classification accuracies in prediction and classification.

### 5.4. Experimental Comparison

#### 5.4.1. Comparison of Different Preprocessing Methods

**Figure 12:**
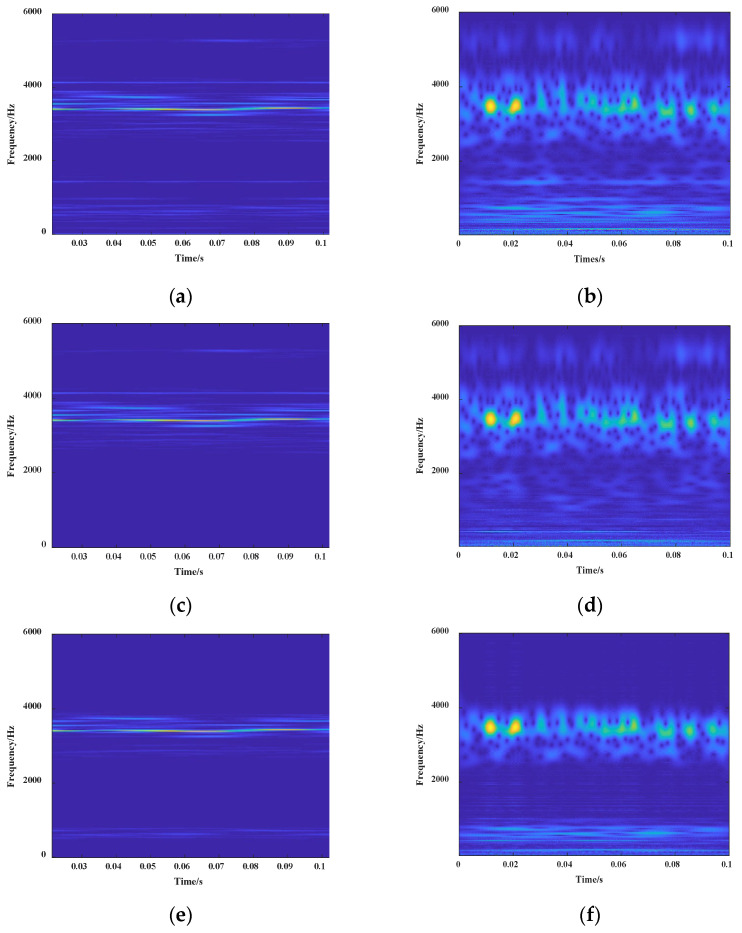
Results of different extraction and processing methods: (**a**) STFT image; (**b**) CWT image; (**c**) EMD and STFT image; (**d**) EMD and CWT image; (**e**) IVMD and STFT image; (**f**) IVMD and CWT image.

In this paper, to verify effectiveness of the proposed method, the IVMD and CWT methods were compared with traditional time–frequency conversion methods such as STFT, CWT, and EMD methods. In traditional fault detection, the one-dimensional fault signal is not processed before being input to the CNN for time-domain feature extraction. The conventional method directly converts the vibration signal into a two-dimensional time–frequency image. Firstly, this paper selected STFT and CWT for comparative analysis. As an example, the time–frequency diagram of the one-dimensional signal of fault 6 converted by these two methods is shown in Figure 12a,b.

This paper compares the improved method of variational mode decomposition with EMD to analyze their extraction effects on time-domain features.

The faults 0–9 were decomposed to obtain a series of decomposed components. The difference between EMD and VMD is that the EMD can automatically match the decomposition number *K* of the mode according to the signal type. For comparison, in this section, the EMD and IVMD were used to preprocess different types of fault signals. Then the relevant components were extracted for signal reconstruction, and the reconstructed signals were subjected to time–frequency transformation by STFT and CWT. As an example, the processing result of fault type 6 according to the above method is shown in Figure 12c,d.

After preprocessing the fault data according to the IVMD method, the STFT was performed on the one-dimensional signal and compared with the CWT. The result after the time–frequency conversion of fault 6 is shown in Figure 12e,f.

**Figure 13:**
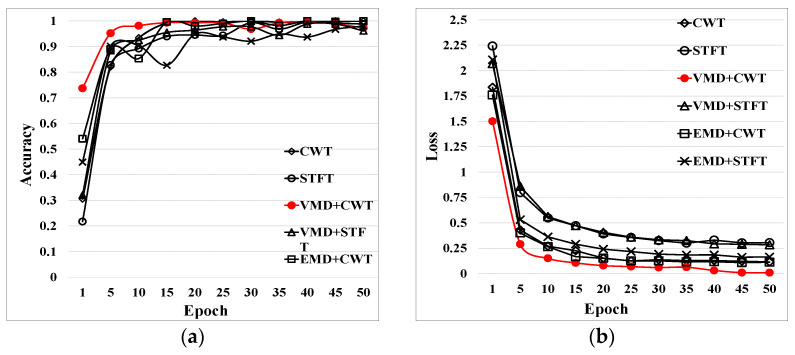
Training comparison results of different methods: (**a**) training accuracy results; (**b**) training loss curves.

It can be seen from the above that different types of fault signals were used for data enhancement preprocessing using various time-domain feature extraction methods. Furthermore, the one-dimensional time-domain signal was converted into a time–frequency-domain image. A training set, a dataset, and a validation set were established for the fault signals processed by each algorithm, respectively. The processed data were set into the established intelligent fault model for identification and classification. The experimental results of 50 iterations of the diagnostic model and the training accuracy curve and loss curve are shown in Figure 13.

It is difficult for the traditional STFT and CWT methods to process non-stationary signals, such as bearing faults. However, EMD is able to process a non-stationary fault signal, extract characteristic components, reduce noise interference, and improve measurement accuracy. The IVMD can adaptively determine the optimal modal components, realize accurate decomposition of fault signals, and avoid the modal aliasing phenomenon generated in EMD decomposition. Therefore, the IVMD method has better accuracy and loss rate than the EMD method. For further investigations, various approaches were used for training and testing. The training accuracy of the diagnostic model for 50 epochs in the validation set and test set is shown in Table 7.

The results of each algorithm after the model training are shown in Table 7. Comparing the two methods for extracting the temporal features from the signal, the IVMD method with automatic optimal decomposition *K* was better than the EMD method. The experimental results show that the proposed method could identify various faults in rotating machinery with a high recognition rate.

#### 5.4.2. Comparison of Different Networks

**Figure 14:**
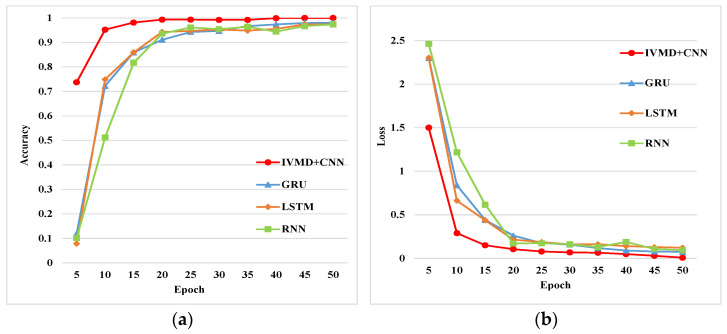
Comparison of the four methods: (**a**) accuracy of different methods; (**b**) loss of different methods.

The IVMD and CNN, GRU, LSTM, and RNN methods were used to train the collected multiple sets of bearing fault data in this paper. The training results are shown in Figure 14.

These deep learning networks were selected for comparison under the same conditions. The traditional recurrent neural network (RNN) could predict continuous data through short-term memory. Long short-term memory (LSTM) is a special RNN, which is mainly used to solve the problem of gradient disappearance and gradient explosion during long sequence training. The input and output structure of GRU is similar to RNN. Compared with LSTM, it has fewer parameters, but it can also achieve the same function as LSTM. The preprocessed one-dimensional vibration fault signals of different types were input into these networks for training. The output feature was 10. The Softmax function was used as the activation function for the output layer in this paper, and the optimizer was Adam. The learning rate of the models in this paper was 0.0001. The batch-size was 50, and the epochs were 50. The loss function of these models was the cross-entropy function. The accuracy of the training results is shown in Table 8.

As shown in Table 8, RNN, LSTM, and GRU were used to predict the time series of fault signals, different fault types could be identified, and then the working state of the rolling bearing could be obtained. However, these networks did not fully extract the time–frequency features of fault signals, and CNN had better feature extraction ability than other methods, so the IVMD and CNN models had higher accuracy.

## 6. Conclusions

Rotating machinery works in a complex environment, and its vibration signals are affected by various excitation sources with nonlinear and non-stationary characteristics. Therefore, this paper presented an intelligent fault diagnosis method based on IVMD and CNN. The proposed method was verified by rotating machinery fault data. Through the analysis of experimental data, the following conclusions were drawn:

(1) It is necessary to pre-set the number of modes in traditional variational mode decomposition. Moreover, the improved variational mode decomposition method in this paper can maximize the difference of segment signals of each adjacent frequency band after decomposition. This method has an acceptable decomposition effect and provides an automatic optimal method for determining the *K* value of the variational mode decomposition.
(2) The main objective of proposing an intelligent fault diagnosis method based on IVMD and CNN is to enhance rotating machinery faults detection. The proposed method has higher accuracy than traditional STFT, CWT, and EMD methods after preprocessing. Furthermore, the proposed IVMD and CNN diagnosis model has higher accuracy than RNN, LSTM, and GRU. Therefore, the end-to-end intelligent diagnosis model has a better recognition and detection effect.

## Figures and Tables

**Table 1:** Parameters table of different fault types.

Fault Types	Fault Diameter (mm)	Length	Label
Normal	0	1000	0
IR	0.1778	1000	1
	0.3556	1000	2
	0.5334	1000	3
OR	0.1778	1000	4
	0.3556	1000	5
	0.5334	1000	6
Ball	0.1778	1000	7
	0.3556	1000	8
	0.5334	1000	9

**Table 2:** Average Pearson coefficient values for different types of faults in the iteration range.

$\begin{array}{cc} & K\\ \mathrm{Peason}\\ \mathrm{Fault}\mathrm{Types} & \end{array}$	5	6	7	8	9	10
0	0.0121	0.0096	0.0130	0.0255	0.0408	0.0569
1	0.0710	0.0798	0.0923	0.0823	0.0806	0.0838
2	0.0815	0.0902	0.0786	0.0778	0.0919	0.0819
3	0.0757	0.0910	0.1050	0.0937	0.0747	0.0932
4	0.1001	0.1497	0.1299	0.1172	0.1161	0.1069
5	0.0846	0.0903	0.0800	0.0867	0.0827	0.0804
6	0.0722	0.0697	0.0662	0.0648	0.0738	0.0748
7	0.0894	0.1146	0.0992	0.0904	0.0983	0.1330
8	0.0712	0.0746	0.0748	0.0761	0.0694	0.0707
9	0.0768	0.0971	0.1201	0.1090	0.1140	0.1183

**Table 3:** Decomposed component correlation coefficient.

*K* = 8	BLIMF1	BLIMF2	BLIMF3	BLIMF4	BLIMF5	BLIMF6	BLIMF7	BLIMF8
Ans	0.2042	0.3646	0.2174	0.3277	0.6725	0.4790	0.2818	0.2114

**Table 4:** Fault 0–9 reconstruction component selection.

Fault Type	Optimal *K*	Reconstructed Components
0	*K* = 6	BLIMF1, 2, 4, 5, 6
1	*K* = 5	BLIMF2, 3, 4, 5
2	*K* = 8	BLIMF4, 5, 6, 7
3	*K* = 9	BLIMF4, 5, 6, 7, 8
4	*K* = 5	BLIMF3, 4, 5
5	*K* = 7	BLIMF4, 5, 6
6	*K* = 8	BLIMF2, 4, 5, 6
7	*K* = 5	BLIMF3, 4, 5
8	*K* = 9	BLIMF4, 5, 6
9	*K* = 5	BLIMF1, 3, 4, 5

**Table 5:** Evaluation indicators comparison for different methods.

Method	*RMSE*	Orthogonal Index
IVMD	0.0461	0.0399
Original VMD (*K* = 5)	0.0467	0.0605
EMD (*K* = 10)	0.0509	0.0436

**Table 6:** The CNN parameters.

Layer Types	Parameters of Layers	Features of the Output
Input layer	3 × 64 × 64 RGB image	3 × 64 × 64
Conv 1	(3, 10, 3, 1)	10 × 64 × 64
Max pooling 1	(2 × 2, 2)	10 × 32 × 32
Conv 2	(10, 40, 3, 1)	40 × 64 × 64
Max pooling 2	(2 × 2, 2)	40 × 16 × 16
Conv 3	(40, 80, 3, 1)	80 × 16 × 16
Max pooling 3	(2 × 2, 2)	80 × 8 × 8
Conv 4	(80, 128, 3, 1)	128 × 8 × 8
Conv 5	(128, 64, 3, 1)	64 × 8 × 8
Max pooling 4	(3 × 3, 1)	64 × 6 × 6
F-C layer 1	(64 × 6 × 6, 1024)	1024
F-C layer 2	(1024, 512)	512
F-C layer 3	(512, num-classes)	10

**Table 7:** Accuracy results of different diagnostic methods.

Different Methods	Accuracy of the Validation Set	Accuracy of the Test Set
STFT	91.43%	96.00%
CWT	93.34%	98.80%
EMD and STFT	90.83%	98.40%
EMD and CWT	95.68%	98.80%
Improved VMD and STFT	93.74%	98.40%
Improved VMD and CWT	97.12%	99.20%

**Table 8:** Accuracy of different networks.

Different Methods	Mean Accuracy
RNN	89.91%
LSTM	90.26%
GRU	91.55%
IVMD + CNN	97.12%

## Data Availability

The experimental data are available from the Bearing Data Center of Case Western Reserve University deposited in http://csegroups.case.edu/bearingdatacenter/home (accessed on 20 April 2022). And the original VMD experimental data are available from the Help Center of MathWorks in https://au.mathworks.com/help/signal/ref/vmd.html?searchHighlight=vmd&s_tid=srchtitle (accessed on 20 April 2022).
